# Validation of the World Health Organization’s Disability Assessment Schedule 2.0 for children with mental disorders in specialized health-care services

**DOI:** 10.3389/fpsyt.2024.1415133

**Published:** 2024-12-18

**Authors:** Lina Díaz-Castro, Miriam Arroyo-Belmonte, Paloma Suárez-Brito, María Elena Márquez-Caraveo, Consuelo Garcia-Andrade

**Affiliations:** ^1^ Direction of Epidemiological and Psychosocial Research, National Institute of Psychiatry Ramón de la Fuente Muñiz, Mexico City, Mexico; ^2^ Independent Researcher, Mexico City, Mexico; ^3^ Research Division, Children Psychiatric Hospital “Dr. Juan N. Navarro”, Mexico City, Mexico

**Keywords:** mental disorders, children, WHODAS 2.0, validity, reliability

## Abstract

**Introduction:**

Mental disorders are highly prevalent among children, yet access to timely and effective treatment remains limited. Untreated or poorly managed mental disorders in children are associated with significant functional deterioration and long-term consequences. The validation of reliable assessment tools is crucial for identifying functional impairments and guiding interventions in this population. This study aimed to assess the utility and psychometric properties of the World Health Organization Disability Assessment Schedule 2.0 (WHODAS 2.0) in evaluating functional impairment among children and adolescents receiving specialized mental health care in Mexican psychiatric hospitals.

**Method:**

A cross-sectional analytical study was conducted from January 2018 to February 2020 in two psychiatric public hospitals in Mexico. The Spanish version of the WHODAS 2.0 was adapted for the pediatric population, and its psychometric properties were evaluated among 390 children and adolescents receiving psychiatric care. Data were analyzed using descriptive statistics, exploratory and confirmatory factor analyses, and tests of internal consistency and validity.

**Results:**

The WHODAS 2.0 demonstrated high internal consistency (α = .92) and convergent validity, with significant correlations observed between WHODAS scores and clinical variables. Exploratory factor analysis revealed a six-dimensional structure, with gender-specific differences identified in functional impairment patterns.

**Discussion:**

The study provides robust evidence supporting the utility and psychometric properties of the WHODAS 2.0 for assessing functional impairment in children and adolescents with mental disorders in Mexican psychiatric hospitals. These findings have implications for clinical practice, policy-making, and future research aimed at improving outcomes for this vulnerable population.

## 1 Introduction

Mental disorders are more prevalent among adolescents and young adults, yet most of these individuals do not have access to timely, effective treatment (1). This lack of access is particularly concerning, as untreated or poorly treated mental disorders in youth are associated with short-term and long-term functional deterioration, including reduced educational and employment opportunities (2).

The disability linked to mental health problems necessitates early identification and intervention to improve long-term prognosis, recovery, and economic outcomes (3, 4). Beyond direct treatment costs, mental disorders lead to various indirect costs, such as expenses associated with conditions exacerbated by disability (4).

To assess level of functioning and disability, the International Classification of Functioning, Disability, and Health (CIF) (5) was developed and validated globally. This multidimensional framework, through the World Health Organization-Disability Assessment Schedule (WHODAS 2.0), groups different domains of health, where the concept of functioning is a broad term that covers bodily functions, activities and participation. WHODAS 2.0 assesses disability, and encompasses deficiencies, activity limitations, and participation restrictions (6). The growing number of studies reflect increased interest in using WHODAS 2.0 to assess individual functioning and disability across various settings and health conditions (7, 8).

WHODAS 2.0 has demonstrated both validity and reliability in assessing individuals with mental disorders (9, 10). It has been widely used in population surveys, clinical practice to monitor patient outcomes, and to evaluate the effectiveness of interventions aimed at reducing disability (11). WHODAS 2.0 has been validated in several high-income countries, including Germany and Portugal (12, 13), as well as in middle-income countries such as China (14). However, few studies have reported on the measurement of functionality and disability among children and adolescents with mental disorders in specialized care settings, particularly in low-resource environments (15). Moreover, there are significant gaps in healthcare access and treatment for mental disorders in youth, due to limited availability of interventions (16), and a lack of data on the degree of functional impairment (14, 15, 17, 18) in specialized mental healthcare services.

The psychometric properties of WHODAS 2.0 have been investigated in adolescent populations. Studies on measurement invariance have been conducted in Canada (19, 20), while reliability and validity have been assessed in low- and middle-income countries, such as rural Rwanda (15) and rural Pakistan (18). Validation efforts included assessments of WHODAS 2.0 among children and adolescents with autism spectrum disorders in Italy (21) and those with chronic physical illness in Canada (22). In clinical settings, functional impairment has been linked to higher rates of mental health comorbidity in children aged 4-17 (23).

The aim of this study was to evaluate the utility and psychometric properties of WHODAS 2.0 scores for assessing functional impairment in children and adolescents with mental disorders receiving specialized care at national psychiatric reference hospitals in Mexico.

We hypothesized that the WHODAS 2.0 would exhibit high internal consistency and convergent validity, with significant correlations observed between WHODAS scores and clinical diagnoses. Additionally, we expected to observe gender-specific differences in functional impairment.

## 2 Methods

### 2.1 Study design and setting

A cross-sectional analytical study was carried out in two psychiatric public hospitals in Mexico. The selection of psychiatric hospitals was guided by rigorous criteria aimed at ensuring representation and relevance to the study’s objectives. Specifically, hospitals were chosen based on their status as national reference psychiatric facilities in Mexico, providing outpatient and inpatient services to children and adolescents without social security coverage and offering subrogated services for adolescents within the social security system. This strategic selection aimed to capture a diverse range of participants accessing specialized mental health care within the Mexican healthcare system.

The study was conducted in two phases. Phase 1 encompassed adapting the Spanish version of the World Health Organization Disability Assessment Schedule (WHODAS 2.0) (18) for the pediatric and adolescent population. Phase 2 evaluated the psychometric properties of the adapted version among children and adolescents receiving psychiatric care services in Mexico.

#### 2.1.1 Phase 1: adaptation of the WHODAS 2.0 for children and adolescents

A team of ten health professionals acknowledged the importance and potential utility of implementing the WHODAS within psychiatric populations. This team comprised one psychiatrist, two psychologists specializing in neurodevelopment, two child psychiatrists, two graduate students specializing in public mental health, and three doctoral-level healthcare professionals (24, 25). Moreover, they highlighted the need to adapt it for use in the pediatric context (26). This team proceeded to adapt the Spanish version of the 36-item WHODAS 2.0, as published by the WHO (17), through a series of three group discussion sessions and piloted its application within the population. In the initial session, the expert group was tasked with rating each item on a scale of one to five in terms of its relevance to the pediatric population and offering suggestions for adaptation where necessary. In a subsequent session, the panel analyzed the potential neurodevelopmental differences between children and adolescents to adapt the items and application instructions of the instrument. In the third session, suggestions and discrepancies identified in the first meeting were deliberated in a plenary session to achieve consensus. The information collected from working documents, meeting notes, and decisions made during the plenary session was analyzed collectively.

Minor discrepancies were identified among the expert team concerning items D4_5 and D3_4, which pertain to sexual activity and the number of days children could be alone. Regarding item D4_5, it was decided to exclude it for children under 12 years old. For adolescents, an initial inquiry would determine whether the question was deemed “applicable” or not. If respondents answered negatively, its application would be omitted. Item D3_4 underwent modification by changing the term “days” to “hours”, resulting in: “Being alone for a few hours”. Additionally, nine items were adjusted to reflect the types of activities typically engaged in by children and adolescents, particularly focusing on school activities instead of work activities and providing examples relevant to the pediatric population. Experts reviewed the application instructions of the WHODAS version administered by a trained interviewer, ensuring it was directed to the child’s primary caregiver or guardian, and made minor adjustments accordingly.

A pilot implementation of the revised version of the instrument was conducted. A checklist was used to assess the clarity and comprehension of the instrument as a whole, as well as each item, the instructions, and the response options. The pilot study was carried out over a two-week period (one week in each psychiatric hospital), involving a total of 20 children and adolescents with characteristics similar to those of the final sample. Overall, participants demonstrated a clear understanding of each item. Following the pilot test, experts held a third session to discuss relevant adjustments, primarily focusing on the application strategy. The instructions were adapted to create a version of the instrument suitable for “Children and Parents”, directly questioning children in the presence of their primary caregiver, who, in the event of any discrepancies, validated or adjusted the child’s response, and only when they reached a consensus, was the response recorded. The cards attached to the interviewer-administered version of WHODAS 2.0 were used. Card 2 was nuanced using colors to represent response levels for easier identification by children: a lighter color for the “None” response option, gradually transitioning to a deeper red for the “Extreme/Cannot do” option. The purpose was to provide children with a visual indication or reminder of response options. Interviewer instructions stipulated throughout the WHODAS 2.0 were followed, indicating when each card should be presented to the interviewee. Questions related to school were not asked to children who were not enrolled in school. The responses from this group of participants were included in the validity analyses.

#### 2.1.2 Phase 2: validation of the WHODAS

A total of 397 children of both sexes between the ages of 5 and 18 who received outpatient’s mental healthcare services at either hospital during January 2018 to February 2020 were selected for the study. A probabilistic sample calculation was performed, to estimate a proportion for a finite population, with a 95% safety margin (27). The list of children for each participating service was consulted and a simple random selection was made to be considered in the study. The primary caregivers of the selected child identified were invited to participate, and acceptance was given to sign the informed consent. The information was captured immediately in Access computer software, through the SQL database, for further analysis.

Interviewers responsible for administering the WHODAS 2.0 (two graduate students in public mental health) underwent comprehensive training in the use of the instrument and the principles of the International Classification of Functioning, Disability, and Health (ICF). This training aimed to standardize data collection procedures and minimize variability in participant responses. Additionally, steps were taken to address missing data through careful monitoring and follow-up with participants to encourage completion of all required information. The interview was carried out in an approximate time of 30 minutes to the primary caregivers (parents or guardians) of the children minor than 12 years old, and to adolescents between 12-18 years old, users in each participant service.

### 2.2 Instruments

#### 2.2.1 The World Health Organization Disability Assessment Schedule-2.0 (Children)

The WHODAS 2.0-Children was composed of 35 items divided into the 6 domains proposed in the original instrument: 1) *Cognition* – understanding and communicating (6 items); 2) *Mobility* – moving and getting around (5 items); 3) *Self-care* – attending to one’s hygiene dressing, eating and staying alone (4 items); 4) *Getting along* – interacting with other people (5 items); 5) *Life activities* – domestic responsibilities, leisure and school, with 4 items for chores and 5 items for school activities; and 6) *Participation* – joining in community activities, participating in society (5 items). Each item prompted children or adolescents to assess the level of difficulty on a five-point scale, ranging from none to extreme/cannot do. After completing the questionnaire, participants were asked to indicate the number of days out of the previous thirty during which they experienced the identified difficulties. The final instrument score was computed using the “simple scoring” method specified in the WHODAS 2.0, where the scores assigned to each question were totaled.

#### 2.2.2 Sociodemographic

Variables such as age, sex, schooling, school years completed, marital status, occupation, and diagnosis.

#### 2.2.3 Psychiatric diagnosis and months of treatment

Psychiatric diagnosis and duration of treatment were determined by reviewing the patient’s latest appointment record. This record includes the current main diagnosis according to the International Classification of Diseases 10^th^ Revision (ICD-10) (28) relying on the child psychiatrist assessment. Information regarding the number of months the patient has been receiving treatment at the healthcare institution was also obtained In cases where this information was not available in the appointment record, the treating physician was consulted directly for clarification.

### 2.3 Data analysis

Item analysis was conducted using the frequency distribution obtained for each item. Skewness and kurtosis scores were also obtained for each item to identify those with values > 2. T-scores were then calculated to compare the high group against the low group for each item and to eliminate those that did not discriminate between the two groups. The instrument’s structure was evaluated through an exploratory factor analysis (EFA) using the principal axis factoring method with promax rotation, and confirmatory factor analysis (CFA) using the maximum likelihood estimation method. Age groups were not used to enable more robust analyses.

The Comparative Fit Index (CFI), Incremental Fit Index (IFI), and Normed Fit Index (NFI) were used to assess the fit of the WHODAS 2.0 structure. Proposed modifications were made based on modification indices to improve the model fit. The internal consistency of each item within each domain and overall was evaluated using Cronbach’s alpha and McDonald’s Omega. Convergent validity was assessed by calculating Pearson correlation coefficients (r) between the total scale and its dimensions with months of hospital care and education. The distribution of scores from resulting dimensions by sex, psychiatric diagnosis and age was also examined. Analyses were conducted using SPSS version 25 and AMOS version 24 (Scientific Software International Inc., Skokie, IL, USA).

## 3 Results

### 3.1 Sample description

A total of 397 children participated in the study. The sample considered for the present study consisted of 390 individuals after excluding cases where extreme responses (outliers) were identified in more than three items through item analysis. The characteristics of the sample by type of diagnosis are shown in **Table 1**.

**Table 1:** Demographic characteristics of participants.

					Diagnosis			
	Total Sample		Depressive disorder		Hypercinetic disorders		Others	
	n=390	%	n=134	%	n=199	%	n=57	%
Sex								
Female	146	37.4	68	17.4	39	10.0	39	10.0
Male	244	62.6	66	16.9	160	41.0	18	4.6
Age								
6-12 years	202	51.8	64	16.4	136	34.6	3	0.8
13-18 years	188	48.2	70	17.9	64	16.4	54	13.8
Occupational activity								
Student	355	91	122	31.3	185	47.4	48	12.3
Inactivity due to health issues	18	4.6	5	1.3	7	1.8	6	1.5
Others	17	4.4	7	1.8	7	1.8	3	0.8
Education (years)		*Mean* *SD*		*Mean* *SD*		*Mean* *SD*		*Mean* *SD*
	390	5.8 +-3.6	134	6.6 +-3.4	199	4.4 +-3.4	57	9.8 +-2.3
Living arrangements of Children								
Parents	361	92.6	117	30	189	48.5	55	14.1
Grandparents	21	5.4	11	2.8	8	2.1	2	0.5
Others	8	2.1	6	1.5	2	0.5	0	0.0
Hospital for Medical-care								
Children´s Psychiatric Hospital	248	63.6	71	18.2	169	43.3	8	2.1
National Institute of Psychiatry	142	36.4	63	16.2	30	7.7	49	12.6

### 3.2 Item analysis

One item (D3_4) was removed from further analysis due to the high frequency of responses in a single category. Skewness values ranged from -0.05 to 1.93, and kurtosis values ranged from -0.96 to 3.16. The difference between the total scores of the low disability group (mean = 1.48; SD = 0.160) and the high disability group (mean = 2.63; SD = 0.436) was significant (p <.001) for all items. The item-total score correlations ranged from 0.30 to 0.59. No items were discarded due to non-committed responses.

### 3.3 Exploratory factor analysis

The EFA revealed a Kaiser-Meyer-Olkin (KMO) measure of sampling adequacy value of.895, and the significance of Bartlett’s test of sphericity was p < 0.001, indicating both were satisfactory. The EFA yielded 6 dimensions with a total of 34 items, where 5 generally aligned with the theoretical dimensions. The dimensions of Daily activities at home and school were separated, while the theoretical dimensions of Mobility and Self-care were combined. The total percentage of explained variance was 50.37%. (**Table 2**) displays the solution and factor loadings for each of the items.

**Table 2:** Exploratory factor analysis of WHODAS-Children.

	Cognition	Mobility and Self-care	Getting along	Life activities: Home	Life activities: School	Participation
D1_1	**.650**	.439	.286	.400	.432	.471
D1_2	**.597**	.416	.308	.298	.346	.420
D1_3	**.619**	.368	.356	.340	.337	.388
D1_4	**.616**	.341	.290	.220	.238	.223
D1_5	**.671**	.407	.402	.261	.310	.332
D1_6	.518	.331	**.531**		.137	.169
D2_1	.390	**.443**	.182	.284	.255	.243
D2_2	.476	**.599**	.270	.248	.233	.248
D2_3	.382	**.695**	.293	.246	.226	.152
D2_4	.321	**.667**	.400	.202	.143	.166
D2_5	.313	**.526**	.307	.237	.127	.259
D3_1	.238	**.553**	.183	.396	.233	.315
D3_2	.284	**.495**	.206	.418	.280	.358
D3_3	.205	**.464**	.277	.275	.233	.337
D4_1	.349	.340	**.759**		.166	.261
D4_2	.388	.402	**.710**	.274	.279	.419
D4_3	.376	.475	**.633**	.291	.289	.356
D4_4	.337	.369	**.884**	.124	.216	.391
D5_1	.311	.380	.140	**.827**	.370	.304
D5_2	.352	.454	.208	**.815**	.348	.335
D5_3	.370	.398	.166	**.900**	.431	.365
D5_4	.414	.378	.194	**.842**	.478	.373
D5_5	.369	.300	.261	.399	**.866**	.352
D5_6	.411	.318	.261	.409	**.888**	.355
D5_7	.383	.298	.213	.399	**.902**	.365
D5_8	.344	.286	.173	.397	**.865**	.335
D6_1	.389	.532	**.563**	.214	.352	.492
D6_2	.334	.472	.524	.189	.318	**.544**
D6_3	.375	.391	.338	.329	.360	**.569**
D6_4	.271	.183	.223	.147	.269	**.573**
D6_5	.216	.251	.386	.157	.198	**.687**
D6_6	.224	.105		.309	.185	**.515**
D6_7	.260	.282	.233	.263	.227	**.681**
D6_8	.264	.433	.481	.195	.264	**.566**

Bold values represent the factor loadings of the items that constitute each dimension of the WHODAS 2.0-Children.

### 3.4 Confirmatory factor analysis

**Figure 1:**
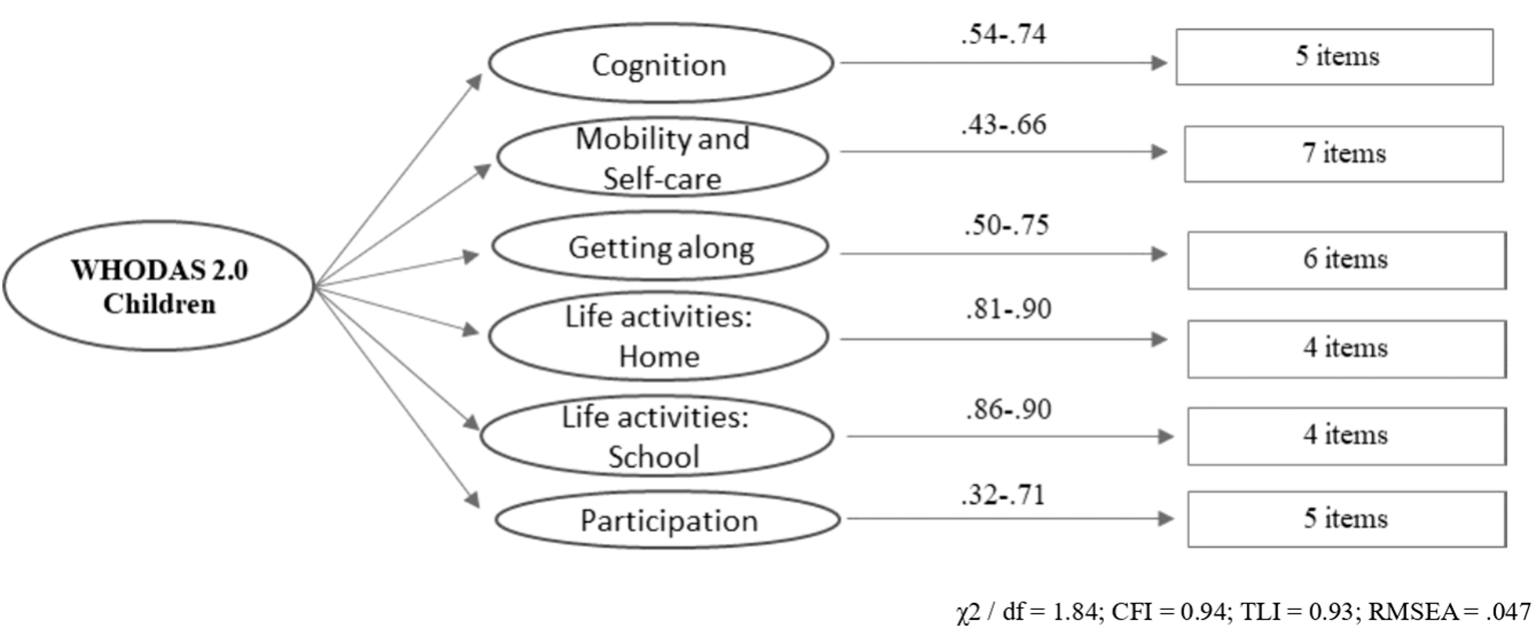
Factor structure of the WHODAS 2.0.

The final model consisted of 31 items across 6 dimensions, each comprising four to seven items (see **Figure 1**). Standardized factor loadings between dimensions ranged from.32 (between Daily Activities-School and Mobility/Self-care) to.76 (between Daily Activities-School and Cognition). Items from the Daily Activities-School domain exhibited the highest factor loadings (0.86-0.90), while items from the Mobility/Self-care domain had the lowest factor loadings (0.43–0.66). There were 3 items with factor loadings below 0.50, two of which were found in the Mobility and Self-care subscale (D2_1 and D3_3), and one in the Participation subscale (D6_8). Goodness of fit indices fell within acceptable ranges (χ2/df = 1.84; CFI = 0.94; TLI = 0.93 and RMSEA = .047).

### 3.5 Internal consistency index

The internal consistency index for the total scale of 31 items was α and ω = .92, with variations among the dimensions from α and ω = .93 for the Activities of Daily Living-School dimension, to α and ω = .75 in the Participation dimension (**Table 3**). For none of the items did the “alpha if item deleted” result in a value greater than that obtained for the total scale.

**Table 3:** Descriptive measures, associated constructs and reliability indices.

	Total	Cognition	Mobility and Self-care	Getting along	Life activities: Home	Life activities: School	Participation
Mean							
Sex							
Female	2.21	2.45	1.76*	2.20**	2.38	2.25*	2.42**
Male	2.12	2.36	1.61	1.98	2.58	2.47	2.15
Age							
6-12 years	2.14	2.40	1.67	1.88**	2.69**	2.49*	2.12*
13-18 years	2.17	2.37	1.67	2.25	2.30	2.71	2.38
Diagnostic							
Depressive disorder	2.14	2.29	1.72	2.07*	2.39*	2.30	2.34**
Hypercinetic disorders	2.13	2.42	1.65	1.97	2.64	2.45	2.05
Others	2.27	2.53	1.63	2.36	2.28	2.35	2.75
Correlation							
**Months of Hospital Care**	-.143**	-.079	-.118*	-.189**	.060	-.057	-.199**
**Education (years)**	0.043	-0.024	0.051	.232**	-0.152**	-0.119*	.141**
WHODAS 2.0-Children							
Cronbach’s Alpha	.917	.782	.753	.837	.910	.933	.746
McDonald’s Omega	.917	.783	.764	.839	.910	.933	.747
Mean^+^	2.16	2.39	1.67	2.06	2.51	2.39	2.25
Standard Deviation	0.59	0.77	0.63	0.86	1.00	1.06	0.82

+ Theoretical Mean = 3; Range: 1 to 5.

**p <.01.

*p <.05.

Hyperkinetic and depressive disorders were chosen as main disorders since they represented 85% (51% and 34%, respectively), the remaining percentage was distributed as follows: Bipolar disorders (0.5%) Schizophrenia (0.2%), Other psychotic disorders (0.5%) Unspecific mental disorders (1.8%) Dissocial behavioral disorders (1.0%) Psychoactive substance use disorders (0.2%) Asperger syndrome (0.5%).

### 3.6 Criterion validity evidence

The criterion validity evidence of the WHODAS was derived from the nomological network, which establishes the various theoretically held relationships of the construct with measurements of other variables such as gender and time of hospital care. **Table 3** describes the means of the total WHODAS-Children scores and its dimensions by these variables. Overall, it is observed that women had higher scores in the Mobility/self-care and Participation dimensions, while men had greater impairment in the Life activities-Home dimension, with no statistically significant differences identified in the total scale. Statistically significant differences were found by age and education in the dimensions of getting along, home, school, and participation, and by type of diagnosis in the dimensions of getting along, home, and participation. Regarding months of hospital care, significant negative correlations were identified in the total scale and the dimensions of Mobility/self-care, Getting along, and Participation.

## 4 Discussion

The findings of our study shed light on critical aspects of mental health care for children and adolescents in Mexican culture, offering insights that hold significant implications for clinical complexity, policy-making, and future research endeavors (29). By evaluating the utility and psychometric properties of the WHODAS 2.0 in assessing functional impairment among this population within the specialized care context of psychiatric hospitals, our study addresses a glaring gap in the literature and provides a foundation for advancing our understanding of pediatric mental health.

Our results contribute to understanding the applicability of WHODAS 2.0 in this context and provide insights into its reliability, validity, and factor structure, adding efforts from other studies (18, 30). High internal consistency (α = .92) across all dimensions demonstrates the reliability of WHODAS 2.0 in assessing functional impairment in this population. The quotient obtained is slightly greater than Scorza’s et al. initial report (2013) of an α = .84 of the child versions of WHODAS 2 in adolescents aged 10-17 and in in agreement with findings in younger children (aged 2-12) with developmental disorders (18) and autism spectrum disorder (21). The CFI and TLI values obtained in this study demonstrate that the structure of the instrument is robust and that the proposed theoretical model is adequately reflected in the empirical data. These results support the construct validity of the instrument in this population, indicating that the measured dimensions are well-represented and consistently align with the underlying theoretical model.

Moreover, our findings demonstrate the instrument’s convergent validity through significant correlations with clinical variables such as psychiatric diagnoses and length of hospital care. These data are in line with Federici et al. (2023) reporting positive correlations with the three DSM-5 levels of impairment and convergent validity with the Autism Diagnostic Observational Schedule (ADOS) in children with autism spectrum disorder (ASD). Our results provide compelling evidence for the utility of the WHODAS 2.0 as a standardized measure for evaluating functional status in young individuals receiving specialized mental health care.

Our findings revealed gender and diagnosis differences in WHODAS scores, aligning with another study, females exhibiting higher levels of impairment in mobility/self-care, getting along and social participation domains, whereas males demonstrated greater impairment in home-related activities (31). Differences in getting along and self-care domains have also been reported between children and adolescents with chronic physical illness have also been reported (22). In our data, girls exhibited greater dysfunction than males in all domains except home and school. This finding is also in agreement with previous reports that emphasize that girls show greater delay compared with boys in perception of psychiatric symptoms, seeking care and first contact with specialized mental health services (32). These gender-specific patterns underscore the importance of considering gender differences in the assessment and treatment of mental health disorders among children and adolescents. Additionally, it is important to note that with respect to timely care, there are differences according to the diagnosis: depressive and anxious disorders have a longer delay between the onset of symptoms and specialized care (32). Barriers to access need to be visualized if functional impairment in children is to be limited (33). On the other hand, childhood trauma has been shown to significantly impact work functioning in adults with mental disorders (34).

Therefore, this instrument emerges as a promising tool offering a multidimensional scale that evaluates various domains of functioning and disability in children, and the findings contribute to a better understanding of the applicability of the WHODAS 2.0 in clinical care settings (30) in the Mexican cultural context. Cultural differences have been reported in other studies (18, 35) and are essential when establishing strategies to enhance access to effective treatments for children with mental disorders.

This work highlights a significant challenge in the field of mental health care for children, which is the scarcity of reports on the measurement of functionality and disability in this population within specialized care services. This gap in the literature is concerning, as it suggests that there may be limited tools available to accurately assess the functional impairments associated with mental disorders in young individuals. Furthermore, the authors point out the broader issue of wide gaps in health care and treatment for mental disorders in children and that such gaps are attributed to a lack of access to existing interventions, insufficient information, and scientific evidence regarding the degree of impairment in their functioning, and a general lack of understanding of how to approach these issues in specialty mental healthcare services (31). Having the WHODAS 2.0 validated in the child population plays a crucial role in bridging the gaps in health care and treatment. By providing a standardized and reliable means of assessing functional impairment, healthcare providers can better identify the specific needs of each child, tailor interventions more effectively, and monitor progress on long-term outcomes of psychopathology (36). Fox example, one research has shown the long-term effects of early-life factors, such as severe acute malnutrition, on functional outcomes in adulthood, highlighting the importance of early intervention (37). Moreover, a validated WHODAS 2.0 could contribute to the accumulation of scientific evidence regarding the extent of functional impairments in this group, thereby informing of this complex field—and is a singular contribution to those who desire to better understand what we know about brain and behavior in children and development of more targeted and effective treatment strategies (38).

### 4.1 Practical implications

The findings on the internal consistency and convergent validity of WHODAS 2.0 in this study highlights its practical value as a reliable tool for assessing functional impairment in children and adolescents with mental disorders within Mexican psychiatric hospitals. By maintaining relevance across developmental stages, WHODAS 2.0 enables clinicians to assess functional disability consistently in both children and adolescents, despite age-related differences. This consistency supports a standardized approach to evaluating key domains of daily functioning -such as mobility, self-care, and social interactions-making it easier to track how mental illness impacts these areas over time. Consequently, WHODAS 2.0 can inform tailored interventions and support plans that respond to individual functional needs, thereby enhancing clinical care and resource allocation. Given that, according to international consensus, WHODAS is part of the standard set of outcome measures for various mental health disorders in children and young people (39), there is an urgent need for researchers and clinicians to contribute to reversing the enormous disease burden of childhood mental disorders (2).

### 4.2 Limitations and future directions

Despite the strengths of our study, including a sizable sample and rigorous psychometric analyses, several limitations should be acknowledged. Future research could explore longitudinal associations between WHODAS scores and clinical outcomes to establish predictive validity. Additionally, cross-cultural validation studies are warranted to confirm the generalizability of our findings beyond Mexican psychiatric hospitals.

## 5 Conclusion

Our study provides robust evidence supporting the utility and psychometric properties of WHODAS 2.0 in assessing functional impairment among children and adolescents with mental disorders in Mexican psychiatric hospitals. By enhancing our understanding of functional impairment domains and their correlates, WHODAS 2.0 facilitates tailored interventions and improves the quality of care for this vulnerable population. Moving forward, it is imperative that researchers, clinicians, and policymakers collaborate to leverage these insights and develop tailored interventions and policies that address the unique needs of young individuals with mental health challenges in diverse cultural and geographical contexts.

## Data Availability

The raw data supporting the conclusions of this article will be made available by the authors, without undue reservation.
